# Oncostatin M Contributes to Airway Epithelial Cell Dysfunction in Chronic Rhinosinusitis with Nasal Polyps

**DOI:** 10.3390/ijms24076094

**Published:** 2023-03-23

**Authors:** Florent Carsuzaa, Emilie Bequignon, Sophie Bartier, André Coste, Xavier Dufour, Matthieu Bainaud, Jean Claude Lecron, Bruno Louis, Stéphane Tringali, Laure Favot, Maxime Fieux

**Affiliations:** 1 Laboratoire Inflammation Tissus Epithéliaux et Cytokines (LITEC), UR15560, Université de Poitiers, F-86000 Poitiers, France; 2 Service ORL, Chirurgie Cervico-Maxillo-Faciale et Audiophonologie, Centre Hospitalier Universitaire de Poitiers, F-86000 Poitiers, France; 3 Centre Hospitalier Intercommunal de Créteil, Service d’Oto-Rhino-Laryngologie et de Chirurgie Cervico-Faciale, F-94010 Créteil, France; 4 CNRS EMR 7000, F-94010 Créteil, France; 5 INSERM, IMRB, Univ Paris Est Creteil, F-94010 Créteil, France; 6 Service d’ORL, de Chirurgie Cervico Faciale, Hôpital Henri-Mondor, Assistance Publique des Hôpitaux de Paris, F-94010 Créteil, France; 7 Service Immunologie et Inflammation, Centre Hospitalier Universitaire de Poitiers, F-86021 Poitiers, France; 8 Hospices Civils de Lyon, Centre Hospitalier Lyon Sud, Service d’ORL, d’Otoneurochirurgie et de Chirurgie Cervico-Faciale, F-69310 Pierre Bénite, France; 9 Faculté de Médecine et de Maïeutique Lyon Sud-Charles Mérieux, Université de Lyon, Université Lyon 1, F-69003 Lyon, France; 10 UMR 5305, Laboratoire de Biologie Tissulaire et d’Ingénierie Thérapeutique, Institut de Biologie et Chimie des Protéines, CNRS, Université Claude Bernard Lyon 1, 7 Passage du Vercors, CEDEX 07, F-69367 Lyon, France

**Keywords:** nasal epithelium, OSM, IL-6, ciliary beating efficiency, epithelial electric resistance, tight junctions, repair rate, CRSwNP, primary culture

## Abstract

Chronic rhinosinusitis with nasal polyps (CRSwNP) is a typical type-2 inflammation involving several cytokines and is associated with epithelial cell dysfunction. Oncostatin M (OSM) (belonging to the interleukin(IL)-6 family) could be a key driver of epithelial barrier dysfunction. Therefore, we investigated the presence of OSM and IL-6 and the expression pattern of tight junctions (TJs) in the nasal tissue of CRSwNP patients and controls using reverse transcriptase quantitative polymerase chain reaction (RT-qPCR) and Western blotting. Then, their potential role in the epithelial barrier was evaluated in vitro in 27 different primary cultures of human nasal epithelial cells (HNECs) by measuring TJ expression and transepithelial electric resistance (TEER) with or without OSM or IL-6 (1, 10, and 100 ng/mL). The effect on ciliary beating efficiency was evaluated by high-speed videomicroscopy and on repair mechanisms with a wound healing model with or without OSM. OSM and IL-6 were both overexpressed, and TJ (ZO-1 and occludin) expression was decreased in the nasal polyps compared to the control mucosa. OSM (100 ng/mL) but not IL-6 induced a significant decrease in TJ expression, TEER, and ciliary beating efficiency in HNECs. After 24 h, the wound repair rate was significantly higher in OSM-stimulated HNECs at 100 ng/mL. These results suggest that OSM could become a new target for monoclonal antibodies.

## 1. Introduction

Chronic rhinosinusitis with nasal polyps (CRSwNP) is a chronic inflammatory disease of the airways characterized by long-term symptoms, such as nasal obstruction and loss of smell, all of which negatively affect patients’ health-related quality of life (QOL) [1] and affect up to 11% of the population of Western Europe [2]. It has garnered considerable public health concern owing to its high incidence and unsatisfactory treatment outcomes. In the latter, 85% of CRSwNP cases reveal a type 2 inflammatory pattern [3], which may contribute not only to the chronic inflammatory state of the sinonasal mucosa but also to tissue remodeling and growth of nasal polyps. The characteristics of airway remodeling include subepithelial fibrosis along with epithelial damage, goblet cell metaplasia, edema, and the loss of differentiation of ciliated cells [1]. The nasal epithelium serves as a mechanical barrier to protect against environmental insults, such as air pollution, viruses, and bacteria, as well as allergens. Patho-physiologically, the damaged epithelium responds to injury caused by environmental factors by inducing the production of Th2-promoting cytokines such as IL-25, IL-33, and thymic stromal lymphopoietin (TSLP) [4]. It has been suggested that the epithelium (in CRSwNP) could be more susceptible to environmental insults due to the inability of nasal polyp epithelial cells to maintain barrier function [5]. To ensure this function, neighboring epithelial cells are connected to one another via tight junctions (TJs) mostly constituted by occludin and zonula-occludens (ZO-1) [6,7]. TJs can be considered gatekeepers whose alteration could contribute to both the aggravation of inflammation-related tissue damage or the resolution of inflammation through drainage [8]. In CRSwNP patients, loss of TJ function and/or expression has been observed [5], and when the epithelial barrier is disrupted, instead of a self-limited immunodefensive response, it results in a chronic inflammatory response that fails to resolve [9,10,11,12].

Although CRSwNP pathophysiology has not yet been clearly identified, chronic type 2 inflammation and epithelial dysfunction appear to be key factors in the formation of nasal polyps, involving numerous cytokines from the interleukin (IL)-6 family [1,13,14,15,16,17,18]. Oncostatin M (OSM) is a multifunctional cytokine belonging to the IL-6 family [18]. OSM has a proinflammatory effect reported on tissues such as the lungs [19], joints, or liver [20] and has the capacity to target skin cells more insensitively than IL-6 [21,22], as described in vitro in primary culture of keratinocytes and in reconstructed human epidermis models [23,24]. OSM protein and OSM mRNA are overexpressed in CRSwNP compared with controls [11] and seem to act both on the fibroblastic and epithelial components of polyp tissues [25] as well as on TJs [11]. IL-6 is mostly regarded as a proinflammatory cytokine, but there are numerous examples of protective and regenerative functions of this cytokine [26]. A recent report demonstrated elevated IL-6 protein levels in the polyp tissue of patients with CRSwNP [27]. In human nasal epithelial cells (HNECs), IL-6 increases ciliary beat frequency and accelerates airway wound repair [28]. However, the involvement of OSM and IL-6 in the pathogenesis of CRSwNP remains largely unknown [25,29].

In the present study, we investigated their potential role in epithelial cell dysfunction in CRSwNP. First, we analyzed IL-6 and OSM expression, the secretion of OSM in outer space, and epithelial TJ expression patterns in nasal polyps. Then, on HNECs at the air-liquid interface (ALI) in vitro, we analyzed (i) the expression pattern of IL-6 and OSM receptors, (ii) the effect of IL-6 and OSM on TJ expression and transepithelial electric resistance (TEER), and (iii) the effect of OSM on ciliary beating efficiency and wound repair capacity.

## 2. Results

### 2.1. In Nasal Polyps, OSM Was Secreted and Overexpressed, and Epithelial TJs Were Altered

**Figure 1:**
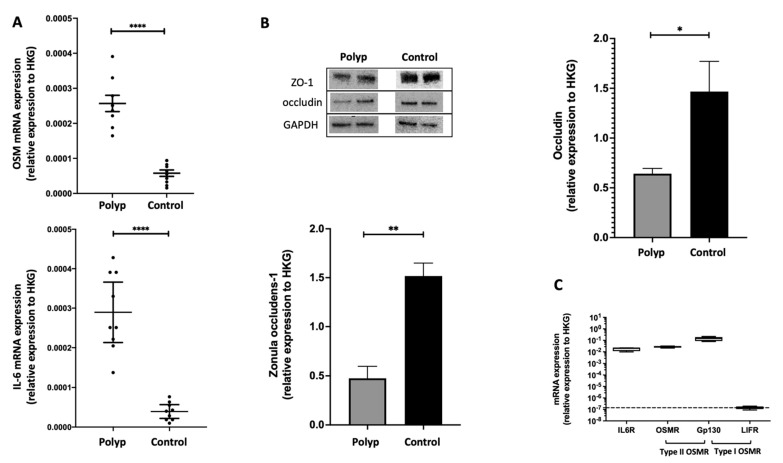
OSM and IL-6 were overexpressed, and epithelial TJs were altered compared to non-inflammatory nasal mucosa. The ex vivo expression of OSM and IL-6 were analyzed by RT-qPCR in nasal polyps and in non-inflammatory nasal mucosa (controls) (n = 9) (**A**) and expression of occludin and ZO-1 were analyzed by Western blotting (n = 5) (**B**), both using GAPDH as a housekeeping gene (HKG) to normalize gene and protein expression. OSM and IL-6 were overexpressed in nasal polyps, and occludin and ZO-1 were underexpressed. The type II OSM receptor and IL-6 receptor were expressed in nasal-polyp-derived HNECs (n = 5) (**C**). The *y*-axis represents the relative expression normalized to HKG. * *p* = 0.0159; ** *p* = 0.0079; **** *p* < 0.0001.

In nasal polyps and in non-inflammatory nasal mucosa, we compared the expression of OSM and IL-6 in RT-qPCR. OSM and IL-6 were overexpressed in the nasal polyps when compared to the control mucosa (*p* < 0.0001) (Figure 1A). 

The presence of OSM in supernatants of 48 h cultured polyps from patients with CRSwNP was assessed by ELISA. The mean OSM concentrations were 192 ± 75 pg/mL [120–290 pg/mL], n = 5 (Figure S1). We also studied by Western blot the ex vivo expression of epithelial TJs occludin and ZO-1, known to be implicated in the human nasal epithelial barrier. We reported a decrease in the expression of occludin (*p* = 0.0159) and ZO-1 (*p* = 0.0079) in nasal polyps when compared to non-inflammatory nasal mucosa (Figure 1B).

### 2.2. The IL-6 Receptor and Type II OSM Receptor Were Expressed in HNEC

To show that OSM and IL-6 can have a direct effect on epithelial cells, we evaluated the in vitro expression of the two interleukin receptors in human nasal epithelial cells. We confirmed the mRNA expression of the IL-6 receptor (comprising IL-6R and gp130 chains) and the type II OSM receptor (comprising OSMR and gp130 chains) but not the type I OSM receptor (comprising LIFR and gp130 chains) in nasal polyp-derived HNECs (Figure 1C).

### 2.3. OSM but Not IL-6 Decreased Occludin and ZO-1 Expression in HNEC

**Figure 2:**
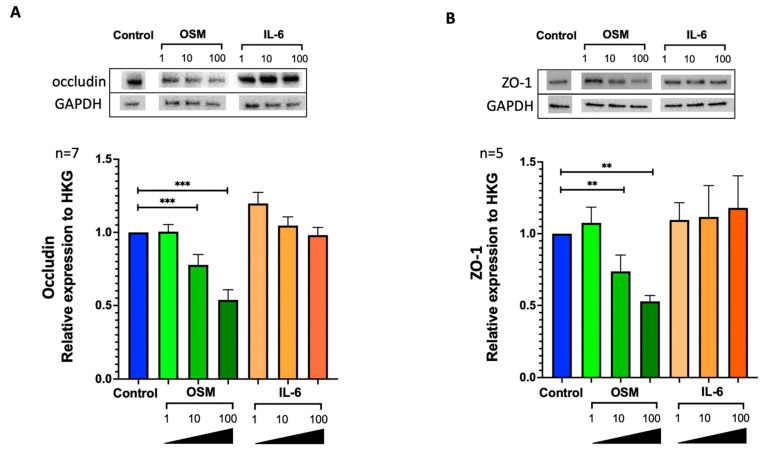
OSM but not IL-6 reduced the expression of occludin and ZO-1 in HNECs. Occludin (n = 7) and ZO-1 (n = 5) relative expression at the protein level were analyzed by Western blotting using GAPDH as HKG to normalize protein expression (**A**,**B**). The *y*-axis represents the relative expression normalized to HKG. Comparisons were made between the groups ** *p* = 0.0079; *** *p* = 0.0006.

**Figure 3:**
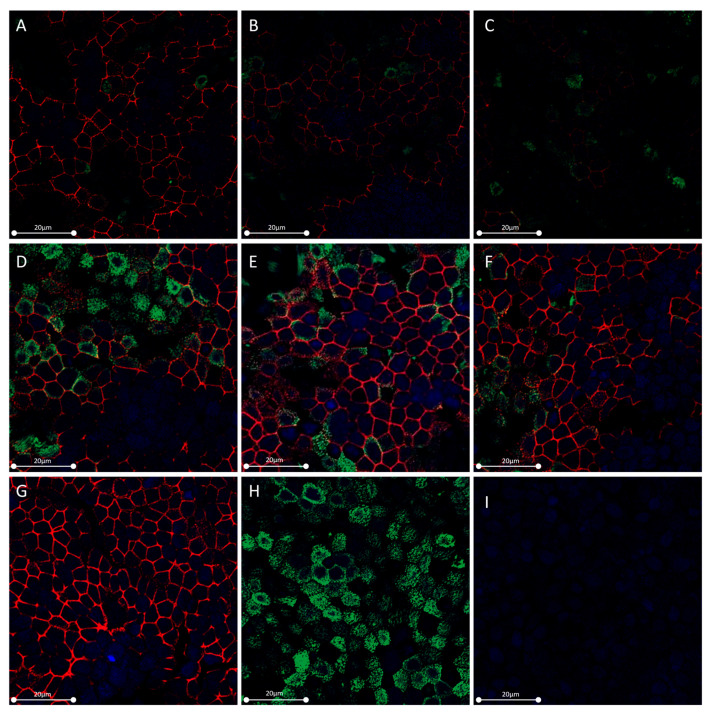
OSM but not IL-6 reduced the expression of ZO-1 in HNECs. ALI cultures of HNECs were stimulated on day 21 with OSM or IL-6 1, 10, and 100 ng/mL for 48 h. ZO-1 (red) and actin (green) expression after immunolabeling and nuclear DAPI labeling (blue) with stimulation by OSM at 1 ng/mL (**A**), 10 ng/mL (**B**), and 100 ng/mL (**C**), and IL-6 at 1 ng/mL (**D**), IL-6 at 10 ng/mL (**E**), IL-6 at 100 ng/mL (**F**), or without any stimulation (**G**,**H**), for ZO-1 and actin, respectively, are shown. A negative control without a primary antibody or rhodamine–phalloidin was performed (**I**).

To evaluate the in vitro effect of OSM and IL-6 on epithelial cell barrier function, we stimulated HNECs with increasing concentrations of OSM or IL-6. In OSM-stimulated HNECs, occludin was underexpressed at 10 and 100 ng/mL (*p* = 0.0006) compared with the control. There was no change in occludin expression compared with the control in IL-6-stimulated HNECs (Figure 2A). Similarly, in OSM-stimulated HNECs, ZO-1 was underexpressed at 10 and 100 ng/mL (*p* = 0.0079), whereas IL-6 had no action when compared with the control (Figure 2B). ALI cultures of HNECs stimulated on day 21 with 10 and 100 ng/mL OSM for 48 h showed a decrease in TJ labeling, whereas no significant changes were observed with IL-6. ZO-1 (red) and actin (green) expression after immunolabeling and nuclear DAPI labeling (blue) with stimulation by OSM at 1 ng/mL (A), 10 ng/mL (B), and 100 ng/mL (C) and IL-6 at 1 ng/mL (D), IL-6 at 10 ng/mL (E), IL-6 at 100 ng/mL (F), or without any stimulation (G and H, for ZO-1 and actin, respectively) are shown in Figure 3. Details regarding actin cytoskeleton organization under the different conditions of the experiment are available in the Supplementary Figures (Figure S2). The actin cytoskeleton organization was the same in the control, under IL-6 for all concentrations tested and under OSM 1 or 10 ng/mL stimulation, whereas OSM 100 ng/mL showed actin stress fibers at the apical pole of the cell.

### 2.4. OSM but Not IL-6 Decreased TEER in HNEC

**Figure 4:**
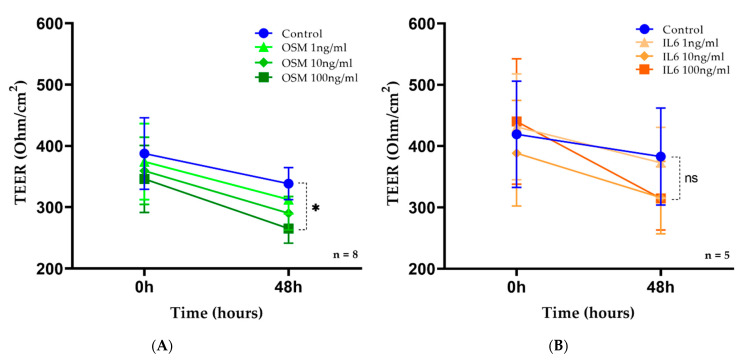
OSM but not IL-6 decreased TEER in HNECs. ALI cultures of HNECs were stimulated on day 21 with OSM or IL-6 1, 10, and 100 ng/mL for 48 h and compared to the unstimulated cultures. TEER measurements in unstimulated controls (blue), OSM 1 ng/mL (light green), OSM 10 ng/mL (green), and OSM 100 ng/mL (dark green), and IL-6 1 ng/mL (light orange), IL-6 10 ng/mL (orange), and IL-6 100 ng/mL (dark orange), were expressed in Ohms/cm^2^. OSM at 100 ng/mL exposure demonstrated a significant decrease in TEER (**A**) whereas IL-6 had no effect (**B**). Data are presented as the change in TEER measurement (Ohm/cm^2^) between the baseline and after the 48 h cytokine exposure. * *p* = 0.049; “ns” indicates *p* > 0.05 between IL-6 stimulated wells and controls.

To evaluate the in vitro effect of OSM/IL-6 on epithelial cell permeability, electrical resistance across the epithelial cell barrier was obtained by TEER measurements with cytokine stimulation (either OSM or IL-6) at various concentrations (1 ng/mL, 10 ng/mL, and 100 ng/mL) and without cytokine stimulation (unstimulated controls). The baseline TEER measurement across all culture wells used for analysis was 367 ± 156 ohms/cm^2^ (mean ± standard error) (n = 8, 81 wells, Figure 4A) prior to OSM exposure, and 430 ± 206 ohms/cm^2^ (n = 5, 39 wells, Figure 4B) prior to IL-6 exposure. Wells with a baseline TEER less than 200 ohms/cm^2^ were excluded. The control well experiments showed a mild decrease in TEER over the 48 h cytokine exposure with TEER at 87.3% of baseline values for OSM stimulation (Figure 4A) and 83.4% of baseline values for IL-6 stimulation (Figure 4B). After 48 h of cytokine exposure, OSM at 100 ng/mL exposure demonstrated a significant decrease in TEER (76.7% of baseline values, 265 ± 68 ohms/cm^2^ at 48 h vs. 346 ± 155 ohms/cm^2^ at baseline, *p* = 0.049) (Figure 4A). No significant result was observed at 48 h for 1 ng/mL (83.5% of the baseline value) and 10 ng/mL (80.7% of the baseline value) OSM or IL-6 at all concentrations tested (Figure 4B) when compared with the unstimulated controls.

### 2.5. OSM Decreased Ciliary Beating Efficiency in HNEC

**Figure 5:**
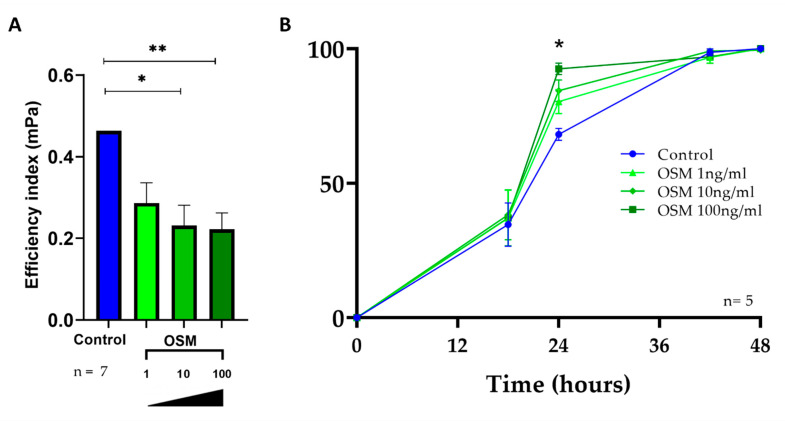
OSM decreased ciliary beating efficiency and increased the in vitro repair rate at 24 h. (**A**) The ciliary beating efficiency (mPa) was evaluated after 48 h of OSM stimulation at various concentrations (1 ng/mL, 10 ng/mL, and 100 ng/mL) and compared with the unstimulated controls (n = 7). Stimulation of HNECs with OSM at 10 and 100 ng/mL decreased ciliary beating efficiency. * *p* < 0.0001, and ** *p* < 0.0001. (**B**) The repair rate was evaluated as the proportion of the wounded area closed at each time point for OSM stimulated wells (either 1 ng/mL, 10 ng/mL, or 100 ng/mL) and compared to one of unstimulated control wells (n = 5). Stimulation of HNECs with OSM at 100 ng/mL increased the wound repair rate. * Indicates statistical significance between the control and OSM 100 ng/mL at 24 h, *p* = 0.016.

The effect of IL-6 on ciliary beating has already been described in a previous study [28]. The in vitro dose-response effect of OSM on ciliary beating efficiency was evaluated in 7 independent primary cultures corresponding to 74 wells (OSM was tested at various concentrations, 1 ng/mL, 10 ng/mL, and 100 ng/mL). After 48 h of stimulation, the mean ciliary efficiency index was 0.433 mPa ± 0.20 in the control wells, and no significant difference was observed with 1 ng/mL OSM (0.324 mPa, *p* = 0.138) (Figure 5A). However, 10 ng/mL and 100 ng/mL OSM significantly reduced the mean ciliary efficiency index when compared with the unstimulated control (0.231 mPa ± 0.19 vs. 0.433 mPa ± 0.20, *p* < 0.0001 and 0.227 mPa ± 0.13 vs. 0.433 mPa ± 0.20, *p* < 0.0001, respectively, Figure 5A).

### 2.6. OSM Accelerated Wound Repair in HNEC

Dysfunction of the repair of the damaged epithelial barrier has been implicated in the pathogenesis of nasal polyps. Our hypothesis was that among the inflammatory cytokines involved in nasal polyps, OSM and IL-6 could alter epithelial repair mechanisms. The effect of IL-6 on wound repair has already been described in a previous study [28]. The in vitro effect of OSM on wound closure was evaluated in 5 independent primary cultures corresponding to 35 wells that were wounded (OSM-1: n = 10, OSM-10: n = 10, OSM-100: n = 10, and unstimulated controls: n = 5). There was no significant difference between the initial wound areas according to each tested condition (219.103 ± 47.10^3^ pixels for unstimulated controls vs. 227.10^3^ ± 35.10^3^ pixels for OSM stimulated wells; *p* = 0.299). As shown in Figure 5B, after 18 h, no significant effect on the wound repair rate was observed. After 24 h, the 100 ng/mL OSM stimulated group had a higher repair rate than the unstimulated control group (90.2 ± 9.5% vs. 72.2 ± 9.6%, *p* = 0.016, Figure 5B). After 44 h and 48 h, no significant effect on the wound repair rate was observed.

## 3. Discussion

In the present study, we first observed that the type II OSM receptor and IL-6 receptor were expressed in HNECs. Then, we found that after IL-6 or OSM stimulation, only OSM had an effect on epithelial barrier function. OSM decreased TJ expression (ZO-1 and occludin), TEER, and ciliary beating efficiency. OSM (such as IL-6 as previously described [28]) also induced dysfunction of the repair mechanism with an increased wound repair rate.

Novel therapies to improve disease control are needed to spare CRSwNP patients from systemic corticosteroids and repeated sinus surgery. Monoclonal antibodies (mAbs) directed against IgE (omalizumab) or cytokines such as IL-5 (mepolizumab) or IL-4/IL-13 receptor (dupilumab) have been tested as innovative therapeutic approaches for CRSwNP. Five randomized, double-blind, placebo-controlled clinical trials demonstrated the efficacy and safety of mAbs [30,31,32]. Improving the knowledge of the different endotypes of CRSwNP should provide insight for determining appropriate current and new therapies [33,34,35,36]. Kim et al. concluded that endotypes depend on epithelial barrier function and epithelial cytokines [34]. Although the effect of IL-4/IL-13 and IL-5 on nasal epithelial repair has already been shown [37], the role of OSM in HNECs has been poorly studied. Pothoven et al. only showed an increase in epithelial permeability with OSM stimulation [11]. In the present study, we chose to investigate the specific role of OSM in HNECs as a potential target for new mAbs. Our hypothesis was that among the inflammatory cytokines involved in nasal polyps, some of the IL-6 cytokine family [18] could alter epithelial repair mechanisms. OSM was selected because it is a pleiotropic cytokine from the same family of IL-6 and has been shown to be more potent than IL-6 in other models, such as skin or liver models [20,23]. IL-6 has been shown to accelerate the wound closure rate in HNECs [28], and OSM could therefore play a key role in the dysregulation of repair mechanisms. We believe that in vitro wound healing is only affected by OSM after 24 h of treatment for two main reasons. First, there was a delay before the healing process started; at 12 h, wound repair was slowly initiated, which made it impossible to see a difference between the cytokine exposures (OSM 1, 10, or 100 ng/mL) and the control wells. Second, after 36 h, all wells were completely healed, including the control. Therefore, only at 24 h was the wound repair rate significantly different between the controls and the 100 ng/mL OSM group. Various in vitro models of epithelial wounding have shown that poorly differentiated cells migrate in the airways without proliferating [38,39,40]. There are two steps described in wound healing: first, proliferation of basal cells, predominant in the wounded area at the beginning, and then, migration and differentiation of epithelial cells leading to wound closure, as shown by Lazard et al. [41]. This process involves attachment and turnover of TJs. Therefore, their alterations impact cell migration and epithelial wound healing [38,39,40]. These results are consistent with our results regarding the wound repair rate after OSM exposure. In nasal polyps, a defective epithelial barrier has been reported with decreased tissue resistance and decreased expression of TJ proteins [5]. A rupture of both the epithelial continuity and the basement membrane is one of the numerous hypotheses underlying the formation of polyps. However, the mechanisms leading to epithelial cell dysfunction remain poorly understood. Extrinsic factors can serve as environmental inflammatory triggers (inhaled irritants or particles, pneumoallergens, commensal and pathogenic bacteria), which induce epithelial damage (barrier dysfunction) and inflammatory mechanisms [1,5,28,42]. In a recent pathogenesis concept, CRSwNP is the result of a chronic nasal inflammatory response triggered by airway epithelial disruption regardless of the initial inductor of aggression [43]. This inductor, such as fungi, bacteria, or lipopolysaccharides (the main outer surface membrane component of Gram-negative bacteria), could increase IL-6 and OSM expression in nasal polyps [16,25]. Upregulation of IL-6 and OSM may also be explained by increased fibroblast activity dependent on ongoing chronic local inflammation possibly initiated by infection [11,25]. Restoring the integrity of the epithelial barrier after injury is also a key element in the defense capabilities of the respiratory epithelium.

TJs establish the polarity of the epithelial cell layer by forming a seal between adjacent epithelial cells, thereby separating the luminal compartment from the basolateral surface [5,8]. We showed that epithelial TJs, mainly represented by occludin and ZO-1, which are crucial proteins in producing the rate-limiting barrier to inhaled pathogens, are decreased in nasal polyps. OSM critically contributes to physiological and pathological processes, including extracellular matrix remodeling, differentiation, inflammatory response, proliferation, and drug resistance [44,45,46]. Therefore, a similar effect on HNECs could be expected, even though it has not been described thus far. The epithelial barrier is sensitive to inflammatory cytokines and surface antigens that alter the distribution of TJ proteins, thereby compromising epithelial barrier function [5]. Multiple chronic inflammatory disease states exhibit epithelial permeability and TJ defects (abnormalities of TJ structure and function), such as asthma and chronic bronchitis [11,47]. The results from experiments on HNECs published by Wise et al. in 2014 support the concept that the CRSwNP epithelium comprises a “leakier” barrier than control mucosae [37]. They also decreased the expression of TJ proteins in vitro in HNECs exposed to IL-4 and IL-13 [37]. These findings support the role of secreted cytokines in the perpetuation of increased epithelial permeability, as we reported with OSM. OSM, secreted following the stimulation of an inductor, amplifies the inflammatory response and is an important factor in the alteration of these TJs. When the HNECs were stimulated by OSM, we noted a decrease in the expression of TJs after treatment with 10 ng/mL OSM. We chose to study the effects of three increasing concentrations (1, 10, and 100 ng/mL) to analyze the dose–response based on our previous in vitro studies on skin epithelial cells [23], demonstrating a dose–response effect. The concentrations chosen are those most frequently used in the literature [11,48,49]. In this study, we did not observe any effect for OSM at 1 ng/mL and a response was observed at 10 ng/mL, similar to the one found at 100 ng/mL. These concentrations are similar to those found in vivo. Therefore, we selected 10 ng/mL as the working concentration. Regarding the expression in tissues, most studies have reported enhanced OSM mRNA, and several studies have evaluated the concentration of OSM secreted by T cells ranging from 0.5 to 10 ng/mL [24,50] demonstrating that the protein is present in the tissue [11,48,49]. In this study, OSM mRNA was enhanced in nasal polyps when compared to the control mucosa; the protein was not detected in the tissue but in the supernatants of the cultured polyps (192 ± 75 pg/mL), as expected for a secretory factor. The question of in vivo relevance is very important, in our study the concentrations chosen were slightly higher, but the response is complex, keeping in mind that cytokines have paracrine effects. In addition, OSM was tested alone and a synergistic effect with other cytokines in vivo should be taken into account. These observations are concordant with those of Pothoven et al., who described an alteration of epithelial TJs with immunofluorescence analysis [11]. Unlike OSM, IL-6 had no effect on epithelial TJs or epithelial permeability. Although IL-6 plays an important role in the development and progression of inflammatory responses, autoimmune diseases, and cancers, as it can induce tissue damage, inflammation, and cell proliferation, IL-6 appears to only act on wound repair and ciliary function in CRSwNP [51,52]. If OSM and IL-6 belong to the same family of cytokines sharing the gp130 receptor, the IL-6 activities were mediated via gp130/gp130 dimerization of the transducing subunits whereas the OSM activities were mediated via gp130/OSMR or gp130/LIFR transducing subunits, recruiting specific downstream signals. This is the reason why, if IL-6 and OSM have closed biological effects, they also have specificities. We previously reported that OSM is clearly more potent and specific on epithelial cells than IL-6 [22], which could explain why OSM but not IL-6 affects the expression of proteins that constitute the TJ. The decrease in TEER in OSM-stimulated HNECs is in agreement with the results reported in normal bronchial epithelial cells by Pothoven et al. [26]. The TEER decrease in the control wells was likely due to manipulation of the ALI cell layer twice in 48 h by the placement of apical media for TEER measurement and subsequent removal of the apical media for continued incubation. The change in basal medium (DMEM-F12) instead of Pneumacult during 48 h to be free of growth factors could be another explanation.

Mucociliary dysfunction is a prominent pathophysiological feature of CRSwNP [53,54,55,56]. After the restoration of barrier integrity following epithelial wounding, mucociliary clearance represents a key step in the defense capacity of the airway epithelium and could be altered in CRSwNP. However, the precise mechanisms underlying mucociliary dysfunction are still unclear. A previous study demonstrated that IFN-γ and IL-13 both significantly reduced ciliated cell differentiation and ciliary beating function in HNECs from CRSwNP patients [54]. In contrast, we previously reported that IL-6 increased ciliary beating functions and metachronal waves without modifying ciliary beating efficiency [28]. Ciliary function is a dynamic process that can be modified by exogen stimuli in physiological conditions [57]. OSM significantly reduced the mean ciliary efficiency index when compared with the unstimulated control, as reported for other proinflammatory cytokines (based on the percentage of ciliated cells and ciliary beat frequency), such as IFN-γ and IL-13 [54]. Regarding the loss of function in HNECs, the ciliary beating index efficiency could be altered secondarily by increased epithelial permeability. If TJs are altered, epithelial barrier function is compromised, and ciliary function is impaired because of cell-to-cell adhesion rupture. The decrease in ciliary function therefore leads to the persistence of pathogens at the apical pole of epithelial cells and the maintenance of the inflammatory state. As OSM also alters TJs, the pathogens that are present can therefore penetrate the chorion and induce a greater inflammatory response. Another hypothesis is the effect of OSM on epithelial–mesenchymal transition (EMT), a crucial process that drives tumor metastasis [58]. OSM reportedly induces EMT and could have the same effect on HNECs. Moreover, in vitro, HNECS are assessed in an air–liquid interface, thus in a liquid medium. The effect of cytokines may be different in vivo from an in situ action in more solid tissue, therefore the dose–response effect transposition is not so evident. A smaller concentration could have a more important effect. To finish, cytokines diffuse in tissues to act in a paracrine way on their membrane receptors. This diffusion has been studied after skin injection of cytokines and could be facilitated on an epithelium whose permeability is increased [24,59]. Targeting OSM could therefore be a therapy of choice by restoring the epithelial barrier and increasing mucociliary clearance.

The repair of epithelial damage occurs in two basic steps. First, basal cells proliferate and migrate into the wound to cover the damaged area; thereafter, the progenitor cells stop proliferating and differentiate into ciliated or goblet cells. Epithelial TJs are then formed to reestablish barrier function in the repaired tissue [11]. Our results showed an alteration of epithelial barrier function under OSM stimulation as described previously by Wise under IL-4 and IL-13 stimulation [37]. Once compromised, the epithelial barrier allows access to the subepithelial tissue, resulting in a chronic inflammatory response [37]. No investigation regarding the potential mechanisms for the accelerated wound repair rate in the OSM exposure group has been performed. Nevertheless, a correlation can be drawn between epithelial permeability and an increased wound repair rate. Indeed, in an IL4-cytokine exposure group from a sinonasal epithelial wound healing model described by Wise et al., the decreased wound repair rate was correlated with an alteration of TJ complex proteins such as annexin A2 [60]. In OSM-stimulated HNECs, the proliferation of epithelial cells is therefore associated with a defective epithelial barrier and an alteration of ciliary function.

In summary, a correlation between the increased wound repair rate and the decreased ciliary beating efficiency could be drawn at a cellular level, but we must note that the molecular basis involved in the process of ciliogenesis remains largely unknown. Laoukili et al. reported that IL-13 alters epithelial cell morphology and ciliated cell differentiation but increases the proportion of secretory cells [55]. Secretory cells and basal cells are involved in cell proliferation in wound healing models. Indeed, epithelial wound repair is characterized by basal cell spreading and migration, followed by proliferation and differentiation. These steps are part of an important process during which epithelial barrier integrity is rapidly restored after a breach. On the one hand, under OSM stimulation, the wound repair rate is increased, probably due to basal cell spreading and migration [61]. On the other hand, under OSM stimulation, the proportion of ciliated cells is reduced, similar to IL-13 stimulation, as reported by Laoukili et al. [55]. The effects reported in this study were enabled through type II OSM receptors, as they were the only OSM receptors identified in nasal polyps. Therefore, we can infer some of the signaling pathways involved with type II OSM receptors for which heterodimerization drives JAK recruitment even though no mechanistic studies have been performed [62]. The C-terminal region of receptor type II contains tyrosine residues, which, phosphorylated by JAK1/2, act as a docking site for STAT1 and STAT3. OSM can also activate other downstream proteins, such as ERK1/2, p38, JNK, the PI3K/Akt pathway, and PKCδ. It can also activate STAT6 in a cell-type-specific way [44,63].

In the present study, we confirmed our hypothesis that OSM alters airway epithelial repair mechanisms by an increased wound closure rate similar to the effect observed with IL-6 on HNECs in a previous study [28] or in other tissues, such as human skin [21,22,23,24]. Elevated OSM may be one signal that initiates a repair step following cellular damage. Studying the in vitro OSM effects on cell differentiation after wound closure could be of great interest. In addition to epithelial cell proliferation, does OSM allow the differentiation of progenitor cells to restore the previous epithelial functions or deleterious functions?

## 4. Materials and Methods

### 4.1. Tissue, Cells Collection, and Primary Culture of HNEC

The nasal polyps and control mucosa were each obtained from nine patients. Small-sized control tissues were obtained from patients operated on for non-inflammatory and non-tumoral diseases of the paranasal sinuses (agreement of the French “Ministère de l’Enseignement supérieur et de la Recherche” (DC-20193746, December 2019)) and frozen in liquid nitrogen before protein extraction. To study OSM secretion by the polyps, the use of control mucosa from healthy patients should be morphologically calibrated on the polyps to be interpretable. During this experiment, the polyps were fragmented into 3 mm^3^ pieces. It appears ethically impossible to take such fragments of mucosa from healthy patients who have not been operated on for CRSwNP or any other infectious and tumoral cause.

HNEC cultures were obtained from nasal polyps from 27 patients undergoing a surgical procedure for CRSwNP. All of the patients gave informed consent and the study was approved by the local ethics committee (CPP IDF X 2016-01-01). All of the patients abstained from oral or topical corticosteroids for one month before the surgery. The tissues were immediately placed in a culture medium and transported to the laboratory for HNEC isolation and culture or frozen in liquid nitrogen before protein and nucleic acid extraction. The HNECs were isolated from nasal polyps as previously described by Coste et al. [12]. Briefly, the nasal polyps were immediately placed in DMEM/F-12 supplemented with antibiotics (100 U/mL penicillin, 100 mg/mL streptomycin, 2.5 g/mL amphotericin B, and 100 mg/mL gentamicin) and sent to the laboratory for processing. Enzymatic digestion [0.1% (wt/vol) pronase in culture medium] was performed for 16 h at 4 °C. The HNECs (1 × 10^6^ cells per well) were then plated in inserts (12 mm Transwell; Costar, MA, USA) with 12 mm-diameter polycarbonate micropore membranes (pore size of 0.4 μm) coated with type IV collagen (Sigma, Saint Quentin Fallavier, France) and incubated at 37 °C in 5% CO_2_. For the first 24 h, the cells were incubated with 1 mL of DMEM/F-12-antibiotics with 2% Ultroser G in the lower chamber and DMEM/F-12-antibiotics with 10% FCS in the insert. After 24 h, the culture medium (Stemcell, Saint Egrève France, Pneumacult-ALI medium) in the insert was removed to place the cells at the ALI. The medium in the lower chamber was then changed every day. The epithelial nature of the cultured cells had already been confirmed by flow cytometric analysis of cytokeratin immunofluorescent labeling showing 95% and 99% of positive cells on days 3 and 7, respectively [64]. The epithelial nature of the cultured cells has previously been well-demonstrated, leading to wide use of the primary culture of HNECs [12,28,41,65,66,67,68,69,70,71]. The HNECs reached a stable differentiated state with the detection of ciliated, secretory, and basal cells during the third week of culture (day 21) by using the ALI culture medium (Stemcell, Pneumacult-ALI medium) [72].

### 4.2. Tight Junctions Protein Expression (ZO-1 and Occludin) and IL-6 and OSM mRNA Expression in the Nasal Polyps and Control Mucosa

To evaluate IL-6 and OSM mRNA expression in the nasal polyps and control mucosa, total RNA from the nasal polyps and control mucosa was isolated using a NucleoSpin^®^ RNA II kit (Macherey-Nagel, Hoerdt, France) and reverse-transcribed with SuperScript^®^ II reverse transcriptase (Invitrogen, Life Technologies, Carlsbad, CA, USA) according to the manufacturer’s instructions. RT-qPCR was performed using a Light Cycler-FastStart DNA MasterPlus SYBR^®^ Green I kit and a LightCycler 480 system (Roche Diagnostics, Meylan, France). The reaction components consisted of 1x DNA Master Mix and 0.5 μM sense and anti-sense oligonucleotides purchased from Eurogentec (Eurogentec France, Angers, France) and designed using Primer3 software. We quantified the expression of OSM (forward: ACTGAGTGCATGAAGCGATG; reverse: CATCGAGGACTTGGAGAA-GC) and IL-6 (forward: CATCCATCTTTTTCAGCCAT; reverse: ATGTAGCCGCCCCACACAGA). Relative RNA expression was determined according to the ΔCT method (relative expression = 2exp(ΔCT) = 2exp (CT target – CT glyceraldehyde-3-phosphate dehydrogenase)) (GAPDH: forward: GGGTGTCGCTGTTGAAGTCAGAGG; reverse: GGCTCTCCAGAACATCATCCCTGC).

To evaluate TJ proteins expression, the nasal polyps and control mucosa were lysed with 350 μL of radioimmunoprecipitation assay buffer (RIPA), and protein concentrations were determined by BCP protein assay (Pierce, Thermo Fisher Scientific, Waltham, MA, USA). After separation on a 4–20% SDS-PAGE gel (NuSep, Germantown, Germany), the proteins were transferred to nitrocellulose membranes (GE Healthcare, Chicago, IL, USA) by electroblotting. Immunodetection of occludin, ZO-1, and GAPDH was performed by co-incubation with a rabbit anti-occludin antibody (Ab) (Thermo Fisher Scientific, Waltham, MA, USA), a mouse anti-ZO-1 Ab (Thermo Fisher Scientific, Waltham, MA, USA) and a mouse anti-GAPDH mAb (Novus, clone 2D4A7), followed by co-incubation with anti-rabbit and anti-mouse IgG peroxidase-conjugated polyclonal Abs (Sigma-Aldrich, St. Louis, MO, USA). Peroxidase activity was detected by chemiluminescence (Luminata HRP substrate from Merck Millipore Burlington, MA, USA) and analyzed using a LAS-3000 imaging system. Signal intensity was measured using ImageJ software v1.53k. The ratios of occludin and ZO-1/GAPDH were calculated and are shown in the corresponding figures.

### 4.3. OSM Secretion by ELISA

To assess the secretion of OSM by nasal polyps, we cultured 3 mm^3^ fragments of polyps from patients with CRSwNP with a culture medium (Dulbecco’s modified Eagle’s medium (DMEM) supplemented with 2 mM L-glutamine, 10% fetal calf serum (FCS), 10 μg/mL gentamycin, 10 μg/mL ceftazidime and 2.5 μg/mL amphotericin B (all from Thermo Fisher Scientific, Waltham, MA, USA).

After 48 h, the supernatant was collected and centrifuged. The detection of OSM was conducted using sandwich ELISA (R&D system, Minneapolis, MN, USA) according to the manufacturer’s instructions.

### 4.4. IL-6 and OSM Receptors RNA Expression in HNECs (RT-qPCR)

On day 21, total RNA from the HNECs was isolated using a NucleoSpin^®^ RNA II kit (Macherey-Nagel, Hoerdt, France) and reverse-transcribed with SuperScript^®^ II reverse transcriptase (Invitrogen, Life Technologies, Carlsbad, CA, USA) according to the manufacturer’s instructions. RT-qPCR was performed using a Light Cycler-FastStart DNA MasterPlus SYBR^®^ Green I kit and a LightCycler 480 system (Roche Diagnostics, Meylan, France). The reaction components consisted of 1x DNA Master Mix and 0.5 μM sense and anti-sense oligonucleotides purchased from Eurogentec (Eurogentec France, Angers, France) and designed using Primer3 software. We quantified the expression of cytokine receptors (IL-6 receptor (IL-6R) (forward: GAGATTCTGCAAATGCGACA; reverse: GTGGGGAGATGAGAGGAACA), OSMR (froward: GGAGACTGCCATTTGCATTT; reverse: GAGTTTGCGGAAAGTGTCAGCAG, LIFR (forward: GCCTGCATTTACAAGGTGGT; reverse: ACCAATGACTGGGCTTTCAC), and gp130 (forward: GGTGACCACTGGGCAATATGACTC; reverse: CGAATGGCAGCATACACAGATGAAG) in the HNECs. Relative RNA expression was determined according to the ΔCT method (relative expression = 2exp(ΔCT) = 2exp (CT target – CT glyceraldehyde-3-phosphate dehydrogenase)) (GAPDH: forward: GGGTGTCGCTGTTGAAGTCAGAGG; reverse: GGCTCTCCAGAACATCATCCCTGC).

### 4.5. ZO-1 and Occludin Protein Expression in HNECs (Western blot) with or without IL-6 or OSM

To evaluate the mechanism of OSM and IL-6 on epithelial barrier function, we evaluated in vitro the expression level of occludin and ZO-1 at protein levels in cytokine-stimulated HNECs. 1 ng/mL, 10 ng/mL, and 100 ng/mL were the concentrations chosen according to previous studies that showed an IL effect on epithelial cells [28,37,60,73] to identify a dose–response effect. After 48 h of stimulation, Western blot was performed as previously described. Immunodetection of occludin, ZO-1, and GAPDH was performed by co-incubation with a rabbit anti-occludin antibody (Ab) (Invitrogen, MA, USA), a mouse anti-ZO-1 Ab (Invitrogen, Waltham, MA, USA), and mouse anti-GAPDH mAb (Novus, clone 2D4A7), followed by co-incubation with an anti-rabbit and an anti-mouse IgG peroxidase-conjugated polyclonal Abs (Sigma-Aldrich, St. Louis, MO, USA).

### 4.6. Actin and ZO-1 Protein Expression in HNECs (Immunolabelling) with or without IL-6 or OSM

After 48 h of incubation at 37 °C and 5% CO_2_ in complete medium (control conditions) or complete medium supplemented with IL (either IL-6 or OSM at 1, 10, or 100 ng/mL final concentrations), once TEER measurements were performed, the cells were washed with PBS and then fixed for 10 min in 4% paraformaldehyde. The first step of immunolabelling was to permeabilize the cells by the addition of 0.3% Triton in PBS Ca^+^ and Mg^2+^ (PBS+/+). Non-specific sites were blocked by incubation of PBS-1% BSA with normal goat serum (PBS-NSA-NGS 1%) for 30 min at room temperature (RT). The cells were thereafter incubated for 1 h at RT with rhodamine–phalloidin reagent (ab235138, Abcam) diluted at 1/2000 in PBS-NSA-NGS 1% for actin fibers (in green). After that, the cells were incubated with an Anti ZO-1 Polyclonal Antibody (Invitrogen) diluted at 1/1000 in PBS-BSA-NGS 1% for ZO-1 expression. ZO-1 was then detected with an Alexa-488 labeled secondary antibody (Life Technology, Carlsbad, California, United States of America, ref. A-21042 diluted 1/1000 in PBS-BSA-NGS 1%). After rinsing with PBS, coverslips were mounted on a slide with the cell side up in ProLong™ Gold Antifade Mountant with DAPI (ref P36935, ThermoFisher Scientific). Stained structures were observed, and images were acquired on an Axio Imager confocal microscope (Zeiss, Oberkochen, Bade-Wurtemberg, Germany) at 63× magnification. As we used the same parameters (labeling intensity and magnifications) for all conditions, qualitative analysis was performed in between conditions.

### 4.7. TEER in HNECs (EVOM) with or without IL-6 or OSM

TEER provides a physical measure of the electrical resistance across the epithelial cell barrier, widely used as an indicator of in vitro permeability [74]. TEER was measured using an epithelial volt-ohm meter (EVOM) with an ENDOHM-12 electrode (World Precision Instruments, New Haven, CT, USA). The values for cell-covered filters were expressed in standard units of ohms per square centimeter (X/cm^2^) after subtracting the resistance of blank filters and are presented as the mean ± standard error. TEER measurements were used before each experiment to assess cellular viability [75] and only HNECs with a TEER > 200 ohm/cm^2^ were used. To evaluate the effect of cytokines on epithelial barrier function, epithelial permeability was determined by measuring TEER in both stimulated and unstimulated HNECs before and after 48 h in various concentrations (1 ng/mL; 10 ng/mL and 100 ng/mL). Cells treated with a medium alone served as a control.

### 4.8. Wound Repair Rate in HNECs (Time-Lapse Images) with or without OSM

The in vitro wound repair assay was carried out according to a model of mechanical injury adapted from a previously described method performed on fully differentiated cultures of HNECs [76]. We created a controlled linear wound of 12 mm length × 1.4 mm by scraping the HNECs with a pipette tip, followed by extensive washing to remove cellular debris (three times with PBS+/+). To determine the repair rate, time-lapse images were taken at regular intervals with an inverted microscope (Zeiss, Axiovert200M, France) equipped with an ×10 objective over a period of 48 h, depending on the time of wound closure (H18, H24, H42, and H48 after wound healing). The wound areas were then quantified by image analysis software (Image J, v1.53k). Wound closure was evaluated as the percentage of wound repair calculating the ratio between the wounded area at each time point and the initial wounded area. The reproducibility of the wound was evaluated by comparing the initial wound area of each concentration tested. Wound closure in the presence of exogenous OSM was compared to the unstimulated control in the context of the serum-free and cytokine-free medium. Immediately after wounding, the HNECs were exposed to DMEM-HAM-F12-Penicillin–Streptomycin–Fungizone–Gentamycin–free medium with or without OSM at various concentrations (1 ng/mL, 10 ng/mL, and 100 ng/mL). Each OSM concentration was tested in duplicate for each culture from the same patient.

### 4.9. Ciliary Beating Efficiency in HNECs (High-Speed Videomicroscopy) with or without OSM

All analyses were performed at controlled RT (20–25 °C). We used an inverted microscope in brightfield conditions associated with a x40 objective. Approximately 30 μL of 4.5 μm polystyrene microbeads 0.125% *w*/*v* was added to 70 μL of survival medium containing beating ciliated cells in suspension and placed between a microscope slide and a cover slide. Cilia movements were recorded with a digital camera (PixeLink A741, Ottawa, ON, Canada) at a rate of 358 frames per second. Each movie was composed of 1800 frames with a definition of 256 × 192 pixels, with each individual pixel being (0.32 × 0.32) μm^2^ with a ×40 objective. All areas containing intact undisrupted ciliated epithelial edges greater than 50 μm, beating in the plane of the camera, were recorded. The efficiency index, a very specific parameter in CRSwNP [77,78], was recorded and analyzed in the ciliated edges of different conditions Each condition was analyzed in duplicate or triplicate for each condition. The ×40 objective was used for all analyses. All of the parameters were studied using the analysis software that we had developed from the Matlab platform (Stream2D). Microbead velocity was used as a marker of the flow generated by the ciliary beating to evaluate the shear stress induced by cilia on the fluid (mean ciliary efficiency index (in mPa)), as previously reported [77,78].

### 4.10. Statistical Analysis

Statistics were performed with R software (v. 4.1.2, R Foundation for Statistical Computing, Vienna, Austria, www.r-project.org, accessed on 1 October 2022). Data were expressed as mean ± standard error of mean (SEM). Comparisons of TJs expression, TEER, ciliary beating, and repair rate between the cytokine-stimulated conditions and the unstimulated controls were performed with a Wilcoxon test (unpaired data and quantitative variables). A *p*-value ≤ 0.05 was considered as significant.

## 5. Conclusions

In summary, we have shown that in vitro, OSM, at concentrations close to those measured in vivo, decreased epithelial barrier function through a decrease in epithelial TJ expression, TEER, and ciliary beating efficiency but with an increased wound repair rate. We posit that OSM dysregulates epithelial barrier function through excessive epithelial wound closure and the loss of ciliary function, leading to a chronic inflammatory state that can evolve toward fibrosis.

Our data suggest that therapeutic inhibition of OSM and its downstream signaling may be advantageous in the treatment of mucosal diseases associated with type 2 inflammation and the loss of barrier function by potentially restoring the barrier function of mucosal epithelial cells.

## Data Availability

Data supporting the reported results can be found in the Laboratoire Inflammation Tissus Epithéliaux et Cytokines (LITEC), UR15560, Université de Poitiers, 86000 Poitiers and in the Laboratoire de l’Institut Mondor de Recherche Biomédical, INSERM U955, CNRS EMR 7000, 93000 Créteil.

## Supplementary Materials

The following supporting information can be downloaded at: https://www.mdpi.com/article/10.3390/ijms24076094/s1.
