# Supplementary material for: Oncostatin M Contributes to Airway Epithelial Cell Dysfunction in Chronic Rhinosinusitis with Nasal Polyps
Source: Int J Mol Sci. 2023 Mar 23;24(7):6094. doi: 10.3390/ijms24076094 (PMC10094365; doi:10.3390/ijms24076094)
Supplement: Supplementary file 1 [file ijms-24-06094-s001.zip › ijms-1980163-supplementary.pdf]

Supplementary Figures

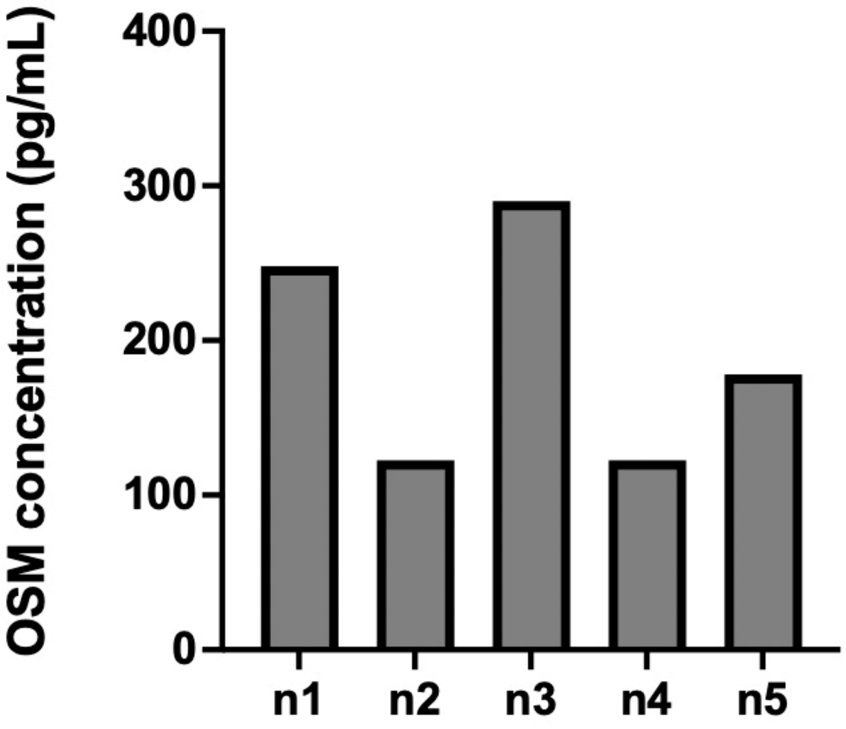

Figure S1: OSM was secreted in cultured polyps

The presence of OSM in supernatants of 48h cultured polyps from patients with CRSwNP was assessed by ELISA. Mean OSM concentrations were 192 +/- 75 pg/mL [120-290pg/mL], n=5

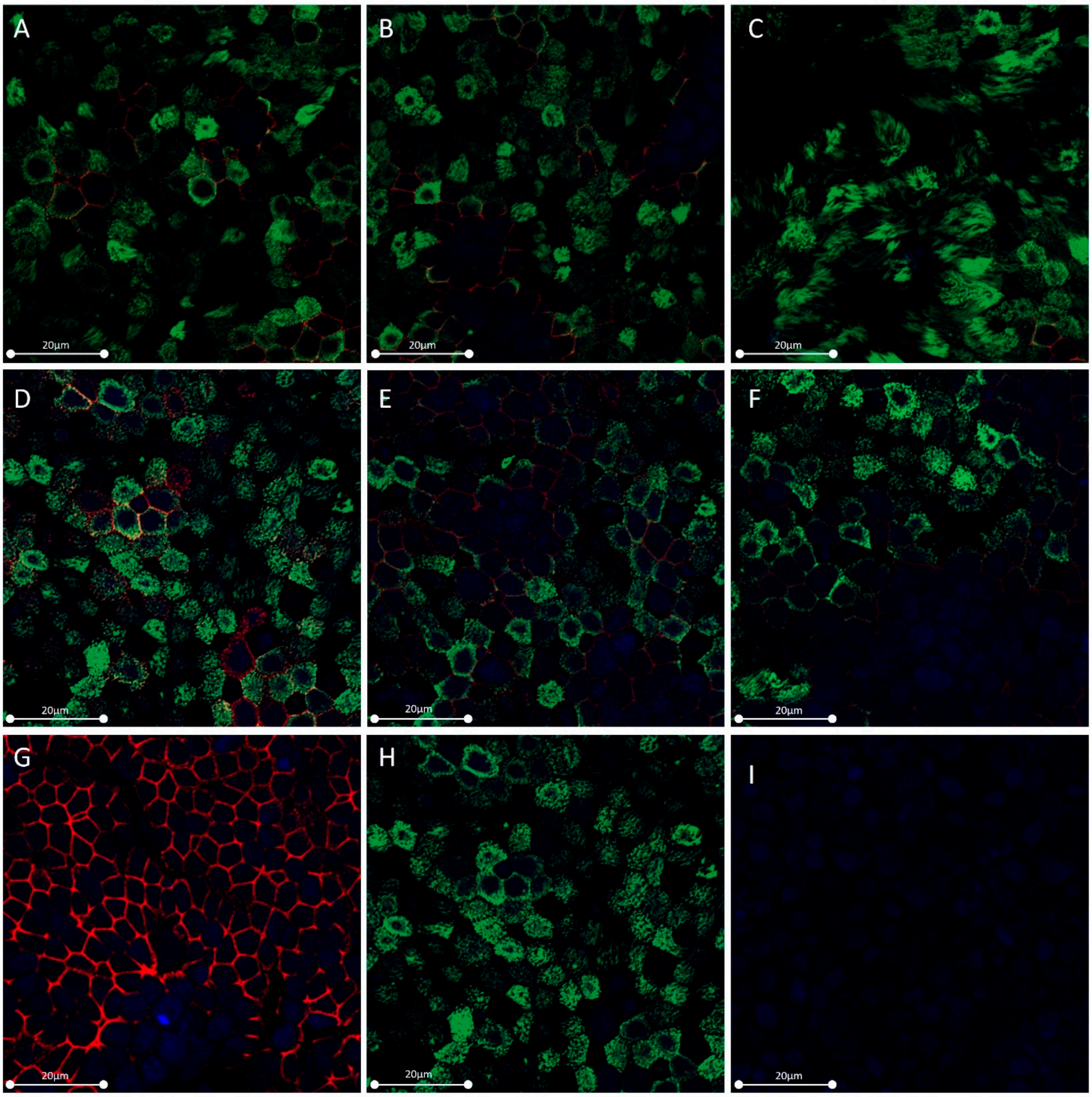

Figure S2: OSM seemed to desorganize actin cytoskeleton in HNEC

ALI cultures of HNEC were stimulated on Day 21 with OSM or IL-6 1, 10 and 100ng/ml during 48 hours. ZO-1 (red) and actin (green) expression after immunolabeling and nuclear DAPI labeling (blue) with stimulation by OSM at 1 ng/ml (A), 10 ng/ml (B) and 100 ng/ml (C) and IL-6 at 1 ng/ml (D), IL-6 at 10 ng/ml (E) IL-6 at 100 ng/ml (F) or without any stimulation (G and H, for ZO-1 and actin, respectively) are shown. A negative control without primary antibody or rhodamine phalloidin was performed (I). The actin cytoskeleton organization was the same in control, under IL-6 for all concentrationsn tested and under OSM 1 or 10ng/ml stimulation, where as OSM 100ng/ml showed actin stress fibers at the apical pole of the cell.
